# Synchronous primary gallbladder and pancreatic cancer associated with congenital biliary dilatation and pancreaticobiliary maljunction

**DOI:** 10.1186/s40792-017-0388-x

**Published:** 2017-11-02

**Authors:** Haruki Mori, Hiroya Iida, Hiromitsu Maehira, Naomi Kitamura, Tomoharu Shimizu, Masaji Tani

**Affiliations:** Department of Surgery, Shiga University of Medical Science, Setatsukinowa-chou, Ootsu, Shiga 520-2192 Japan

**Keywords:** Congenital biliary dilatation, Gallbladder cancer, Pancreatic cancer

## Abstract

**Introduction:**

Synchronous double cancer of the gallbladder and pancreas that is associated with congenital biliary dilatation (CBD) and pancreaticobiliary maljunction (PBM) is extremely rare. PBM is frequently reported in Asia, particularly in Japan. We report a surgical case of synchronous double cancer in a patient with primary gallbladder and pancreatic cancer.

**Presentation of case:**

A 72-year-old woman with epigastralgia underwent subtotal stomach-preserving pancreaticoduodenectomy and gallbladder bed resection for synchronous primary gallbladder and pancreatic head cancer. Histopathological examination revealed moderately differentiated ductal adenocarcinoma of the pancreatic head and well-differentiated tubular adenocarcinoma at the bottom of the gallbladder.

**Conclusion:**

Synchronous gallbladder and pancreatic cancer is extremely rare. It is necessary to determine the optimal surgical course taking into consideration the degree of tumor progression. This is the second case of synchronous primary gallbladder and pancreatic cancer associated with CBD accompanied by PBM.

## Background

Congenital biliary dilatation (CBD) is a congenital malformation involving both extrahepatic bile duct dilatation and pancreaticobiliary maljunction (PBM) [1].

It is well known that PBM is frequently associated with carcinoma of the biliary tract [2, 3]. PBM includes abnormal connection between the pancreatic duct and the common bile duct outside of the duodenal wall, which leads to the reciprocal reflux of pancreatic juices and bile. The normal intra-pancreatic pressure is higher than in the bile duct [4], and pancreatic juice reflux into the biliary tract is confirmed by the presence of activated pancreatic enzymes amylase and lipase [5]. It is thought that the reflux of pancreatic juice into the bile duct and the repeated cycle of biliary epithelium breakdown and regeneration leads to carcinogenesis [6, 7]. Abnormal expression and/or mutation of some oncogenes and cancer suppressor genes occurs during each step of carcinogenesis [8]. Continuous chronic inflammation causes mutations in genes, such as K-ras and p53, that are associated with carcinogenesis and gallbladder cancer [9–11]. As a result, patients with CBD have a high rate of biliary tract cancers. In Western countries, the rate of biliary tract cancer concurrent with CBD is 20%, but this rate is based on very few cases (*n* = 20) [12]. In Japan, a large-scale survey of 2561 patients was undertaken that looked at the incidence of biliary tract cancer concurrent with CBD. The survey found that biliary tract cancer occurs in 21.6% of adult patients with CBD [13]. The main malignancies are gallbladder cancer (62.3%), bile duct cancer (32.1%), and gallbladder plus bile duct cancer (4.7%), indicating that gallbladder cancer is the most frequently found in association with this condition [13]. On the other hand, the evidence for carcinogenesis in pancreatic carcinoma associated with PBM is still lacking. Furthermore, there has been only one other published case of synchronous double cancer consisting of gallbladder cancer and pancreatic cancer associated with CBD. We report herein an interesting and rare case of the above.

## Case presentation

In June 2016, a 72-year-old woman was admitted to the hospital complaining of epigastralgia; however, physical examination did not reveal any abnormalities. Her laboratory tests revealed a normal complete blood count and normal liver function. The serum carcinoembryonic antigen level was 9.3 ng/mL (normal range, < 5 ng/mL), the carbohydrate antigen 19-9 (CA19-9) level was 1084 U/mL (normal range, < 37 U/mL), and the duke pancreatic monoclonal antigen type 2 (DUPAN-2) level was 140 U/mL (normal range, < 150 U/mL). The patient had no history of pancreatitis, pancreatic stone, and cholangitis.

**Fig. 1:**
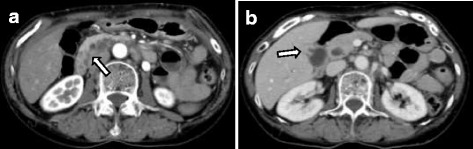
Abdominal computed tomography (CT). **a** Low-density mass and cystic lesions in the pancreatic head. **b** Thickness in the wall adjoining the bottom of the gallbladder

**Fig. 2:**
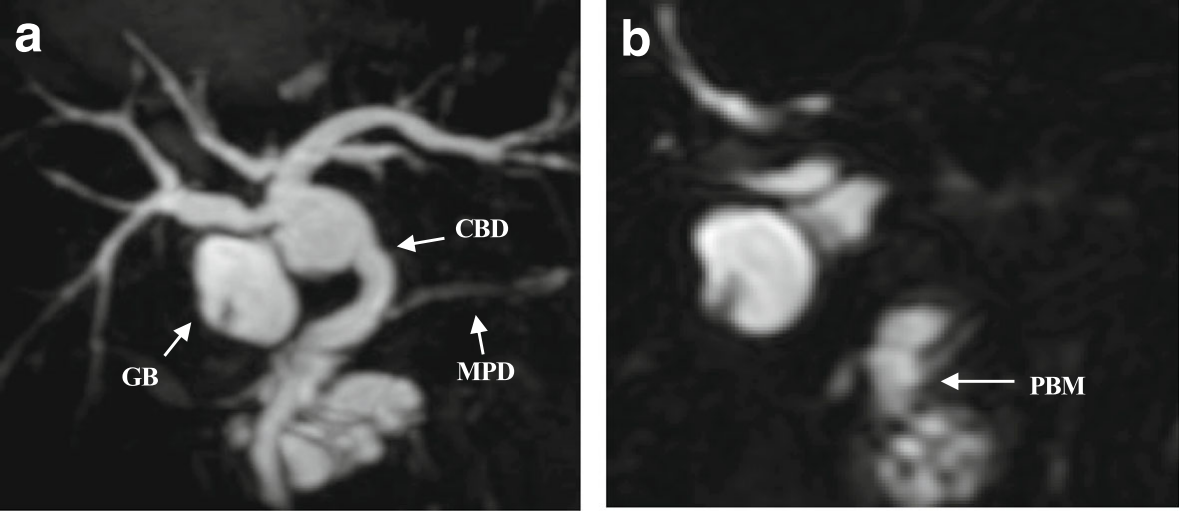
**a**, **b** Magnetic resonance imaging (MRI) showed a pancreaticobiliary maljunction with cystic dilatation of the common bile duct (type Ia [Todani classification]). GB gallbladder, CBD common bile duct, MPD main pancreatic duct, PBM pancreaticobiliary maljunction

Computed tomography (CT) showed a low-density mass in the pancreatic head and thickness in the wall adjoining the gallbladder (Fig. 1a, b). Furthermore, lymph nodes around the pancreas were swelling and multiple cystic lesions with a mural nodule were found in the head of the pancreas. Magnetic resonance imaging (MRI) showed a partially cystic dilatation of the common bile duct, 32 mm in diameter, revealing that the pancreatic duct joined the common bile duct 24 mm above the papilla of Vater. The cystic dilatation of the common bile duct was classified as type Ia using the Todani system [14], and a cystic lesion in the head of the pancreas was confirmed as a branch duct-type intraductal papillary mucinous neoplasm (IPMN) (Fig. 2a, b). Positron emission tomography (PET)-CT showed an abnormal accumulation of 18-fluorodeoxyglucose (FDG) in the pancreatic head and gallbladder. The patient rejected invasive examination: therefore, we did not performed endoscopic ultrasonography (EUS) or Endoscopic retrograde cholangiopancreatography (ERCP) preoperatively.

**Fig. 3:**
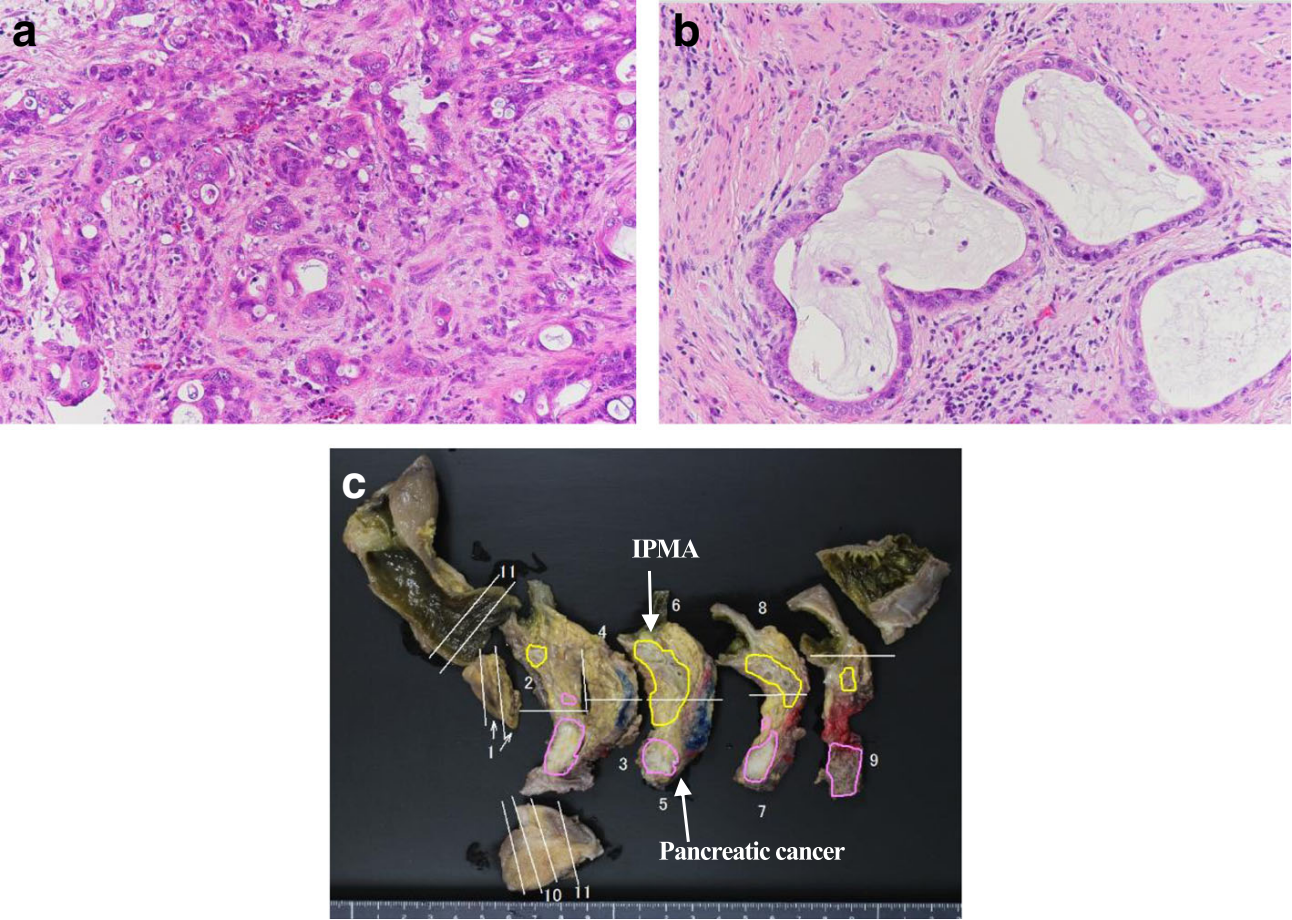
Histological examination. **a** Moderately differentiated ductal adenocarcinoma of the pancreas. **b** Well-differentiated tubular adenocarcinoma of the gallbladder. **c** There was no continuity in histopathological examination between pancreatic cancer and IPMA

The patient was diagnosed with synchronous primary gallbladder and intraductal papillary mucinous carcinoma (IPMC) because a 9-mm mural nodule was found in part of the cystic lesion by CT. Subtotal stomach-preserving pancreaticoduodenectomy and gallbladder bed resection were performed. The patient’s postoperative course was complicated by a grade B pancreatic fistula, as graded by the International Study Group of Postoperative Pancreatic Fistula (ISGPF) criteria, which was treated by percutaneous drainage. The patient was discharged on the 47th postoperative day. Histopathological examination revealed moderately differentiated ductal adenocarcinoma (2.1 × 1.8 cm) in the pancreatic head with retroperitoneal and plexus nerve invasion (Fig. 3a), well-differentiated tubular adenocarcinoma (1.2 × 0.9 cm) at the bottom of the gallbladder with liver serosal invasion (Fig. 3b), and intraductal papillary mucinous adenoma (IPMA) of the pancreas. There were signs of lymphovascular invasion and perineural invasion in the both pancreatic and gallbladder cancer. The cystic lesion detected preoperatively was match to the IPMA in histopathological examination. Concomitant pancreatic cancer did not originate from the IPMA. There was no continuity in histopathological examination between pancreatic cancer and IPMA (Fig. 3c). A total of 16 lymph nodes were harvested and examined. Lymph node metastases were detected in two of infrapyloric lymph nodes, three of lymph nodes along the common hepatic artery, two of lymph nodes in the hepatoduodenal ligament, two of lymph nodes along the superior mesenteric vein, and one of the lymph nodes on the anterior surface of the pancreatic head. However, lymph node metastasis was observed and it was not known whether it originated from the pancreatic cancer or gallbladder cancer. Upon these findings, the stage of pancreatic cancer was pT3N1M0 (pStage IIB), and the gallbladder cancer was pT3N1M0 (pStage IIIB).

Postoperative adjuvant chemotherapy was not performed because of the anticipated impact on quality of life after surgery. Eight months after surgery, she died due to peritoneal dissemination recurrence.

### Discussion

CBD is thought to occur in the development of PBM; however, the pathogenesis of CBD is unknown [1]. The incidence of CBD appears to be higher in Oriental than in Occidental populations [15]. Approximately, one in every 1000 persons is affected by this disease in Japan [16]. In Western countries, CBD occurs in one of every two million births and in every 50,000−150,000 individuals [17–19].

It is important to diagnose bile duct dilation taking into account bile duct diameter, dilated site, and expanded form. Since bile duct diameter varies with age, it is necessary to refer to the upper limit value of the bile duct diameter by age [20]. In our case, the diameter of the bile duct was 32 mm, which extended beyond the upper limit of age.

Todani et al. [21] reported that the reflux of bile may activate pancreatic enzymes, which can cause chronic inflammation and metaplastic epithelial change in the pancreatic duct, and pancreatic cancer may eventually develop. In our case, pancreatic ductal adenocarcinoma developed independently of IPMN in the pancreatic duct, which is an important consideration for future cases. Branch duct type IPMN (BD-IPMN) is associated with concomitant pancreatic carcinogenesis [22–24]. However, the exact diagnosis between pancreatic cancer derived from IPMN and concomitant pancreatic cancer with IPMN is difficult, when IPMN is close to pancreatic cancer histopathologically. Yamaguchi et al. [24] reported that approximately one third of pancreatic cancers derived from IPMN are mucinous carcinoma, and most pancreatic cancers concomitant with IPMN are tubular adenocarcinoma, which are similar to ordinary pancreatic cancers.

We reviewed literatures in PubMed using the terms “pancreatobiliary maljunction,” “congenital biliary dilatation,” “pancreatic cancer,” and “gallbladder cancer” until 2017. To the best of our knowledge, only four cases of PBM with synchronous gallbladder and pancreas cancer have been reported worldwide (Table 1) [3, 25–27]. Four out of five cases were females and presented with CBD. Furthermore, our case is the second to present with synchronous double cancer and CBD. Three cases including ours underwent enlarged cholecystectomy in addition to pancreatectomy. Due to anatomical features, gallbladder cancer develops under the serous membrane in the hepatoduodenal mesoderm and infiltrates beyond the muscularis propria. It directly invades the liver across the liver bed through the cystic duct in advanced cancer with extracapsular invasion; various local progression into the duodenum and/or colon is also observed. In our case, we performed gallbladder bed resection in addition to subtotal stomach-preserving pancreaticoduodenectomy. This procedure has possibility to decrease the quality of life; therefore, the patient could not receive postoperative adjuvant chemotherapy in our case. When choosing the degree of hepatectomy for gallbladder cancer, it is necessary to select the best surgical procedure considering the progression of the tumor and the general condition of each patient.

**Table 1:** Characteristics of reported patients with pancreatobiliary maljunction and double cancer of the pancreas and gallbladder

	Author	Year	Age/sex	Diagnosis	CBD	PBM	Surgery	Survival time (month)
1	Ueda [25])	1992	58/M	Synchronous (GBC, PC, BDC)	–	+	TP Extended cholecystectomy	30 months
2	Minami [3])	2008	50/F	Metachronous (GBC then PC)	+	+	PD	78 months
3	Lahmar [26])	2010	68/F	Metachronous (GBC then PC)	+	+	PD	12 months
4	Rungsakulkij [27])	2013	46/F	Synchronous (GBC, PC)	+	+	PD Extended cholecystectomy	12 months
5	Our case	2016	72/F	Synchronous (GBC, PC)	+	+	SSPPD Extended cholecystectomy	8 months, death

*BDC* bile duct cancer, *GBC* gallbladder cancer, *F* female, *M* male, *PC* pancreatic cancer, *PD* pancreticoduodenectomy, *SSPPD* subtotal stomach-preserving pancreaticoduodenectomy, *TP* total pancreatectomy, *CBD* congenital biliary dilatation, *PBM* pancreaticobiliary maljunction

## Conclusions

In summary, pancreatic carcinoma associated with PBM is a rare event. PBM is closely related to chronic pancreatitis, but the actual relationship between pancreatic carcinoma and CBD accompanied by PBM is unclear, due to lack of sufficient data [3, 26]. Funabiki et al. [28] reported that the incidence of pancreatic cancer is higher in patients with PBM than in patients without PBM. They suspected that the reflux of bile into the pancreatic duct may cause chronic inflammation and cancer of the pancreas. The frequency of pancreatic cancer with PBM is 0.8%, which is relatively lower than biliary tract cancer. However, the incidence of pancreatic cancer in Japan is 16.2 per 100,000 of the population; therefore, 0.8% is 49.4 times higher than the carcinogenic risk in the general population [29]. The present case suggests that patients with CBD accompanied by PBM should be monitored for synchronous cancer of the pancreaticobiliary system and the appropriate surgical procedure should be selected on a per patient basis.
